# Supplementary material for: Dietary Protein Restriction Improves Metabolic Dysfunction in Patients with Metabolic Syndrome in a Randomized, Controlled Trial
Source: Nutrients. 2022 Jun 28;14(13):2670. doi: 10.3390/nu14132670 (PMC9268415; doi:10.3390/nu14132670)
Supplement: Supplementary file 1 [file nutrients-14-02670-s001.zip › nutrients-1751807-supplementary.pdf]

CONSORT Flow Diagram

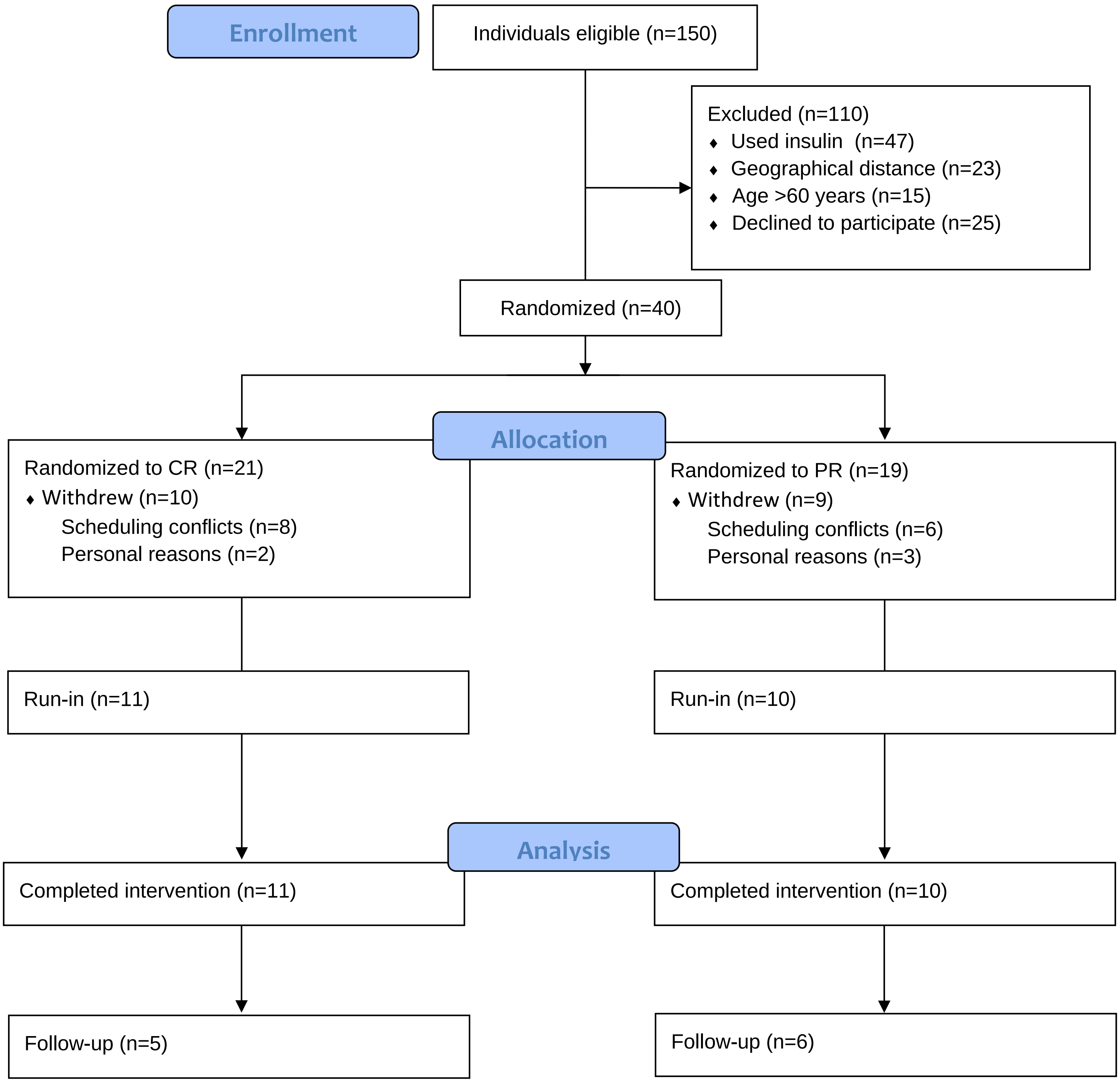

Supplementary Figure S1. CONSORT Flow Diagram

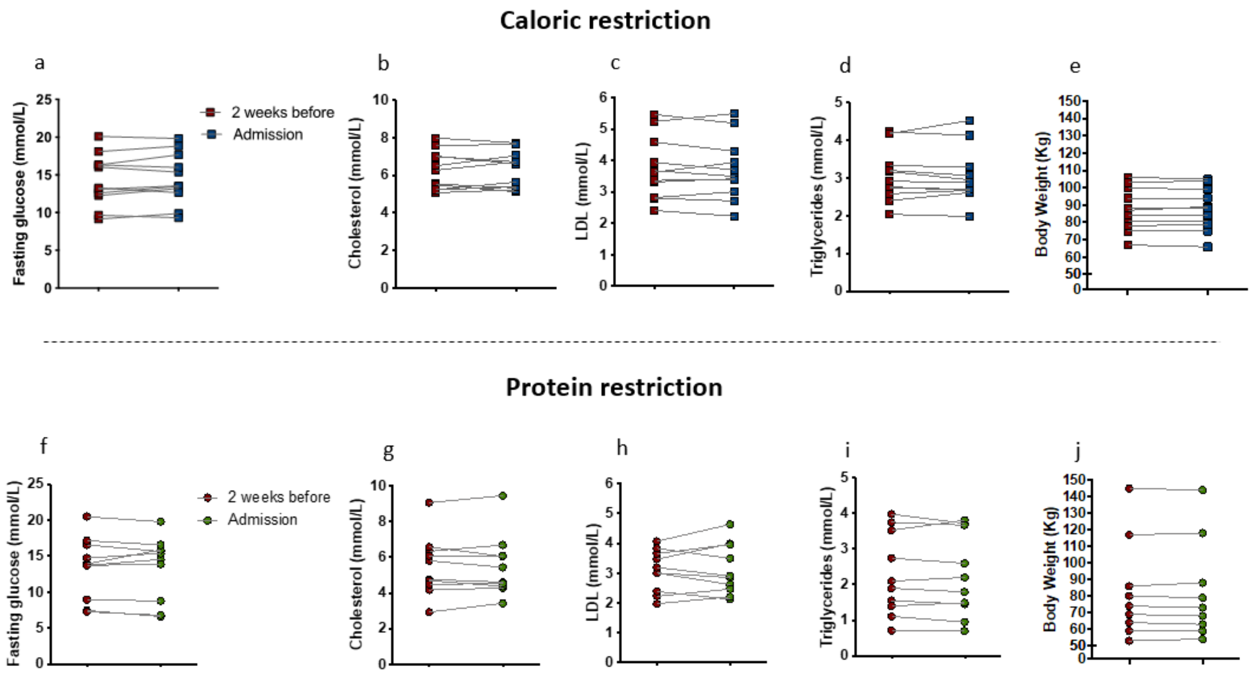

Supplementary Figure S2. Metabolic parameters during run-in period. A-E, individuals later assigned to the CR group. F-J, individuals later assigned to the PR group. **A**, Fasting glucose levels (*P*= 0.2587). **B**, Total cholesterol levels (*P*= 0.8434). **C**, low density lipoprotein (LDL) cholesterol levels (*P*= 0.6301). **D**, Triglycerides levels (*P*= 0.9836). **E**, Body weight (*P*= 0.7560). **F**, Fasting glucose (*P*= 0.9703). **G**, Total cholesterol levels (*P*= 0.9813). **H**, low density lipoprotein (LDL) cholesterol levels (*P*= 0.7136). *I*, Triglycerides levels (*P*= 0.4734). **J**, Body weight (*P*= 0.5911). Data are presented in dot-plot format. CR (n=11). PR (n=10). Paired t test was performed.

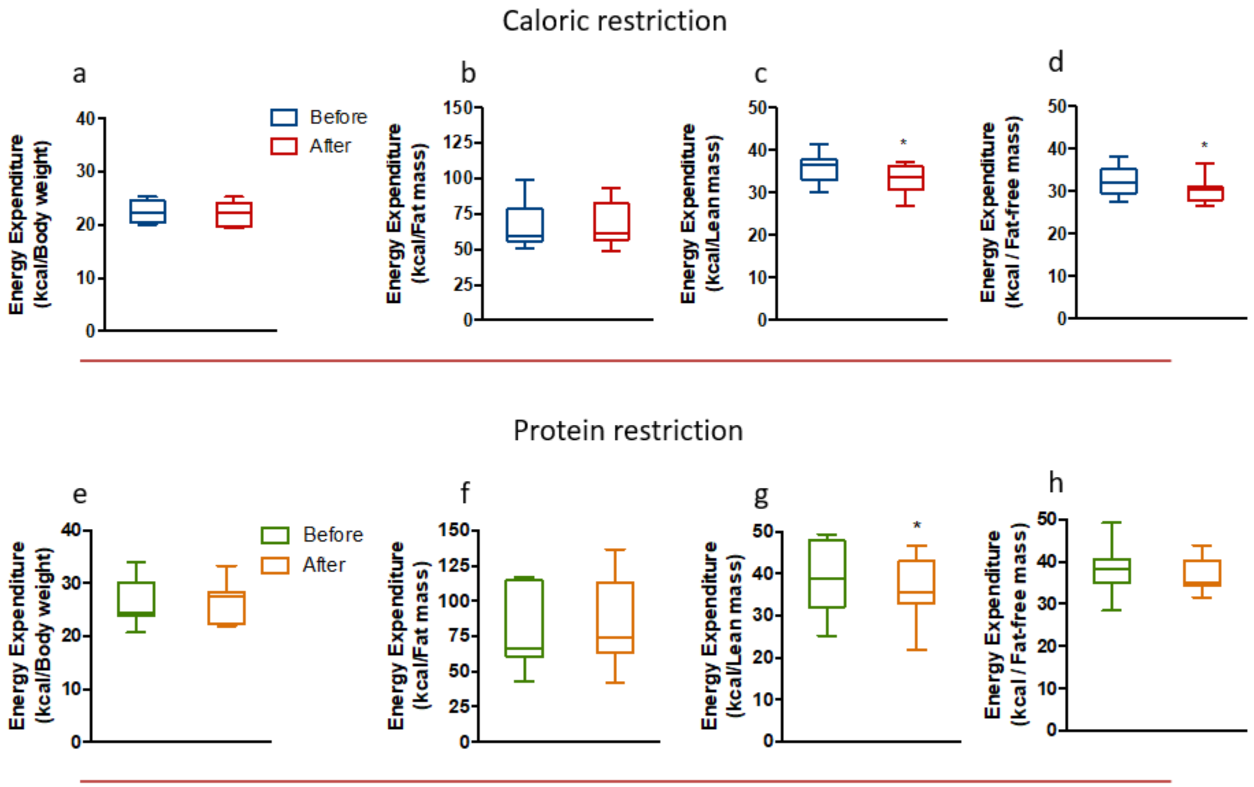

Supplementary Figure S3. Energy expenditure after caloric restriction (CR) or protein restriction (PR). Energy expenditure at rest was measured and normalized by different parameters. (A-D) Comparison after and before CR. A, normalized by body weight (*P*= .7248). B, normalized fat mass (*P*= .9299). C, normalized by lean mass (**P*= .0351). D, normalized by fat-free mass (**P*= .0425). (E-H) Comparison after and before PR. E. normalized by body weight (*P*= .7226). F, normalized by fat mass (*P*= .3707). G, normalized by lean mass (**P*= .0359). H, normalized by fat-free mass (*P*= .1487). Data are shown in box plots (line at median). Paired t test was performed.

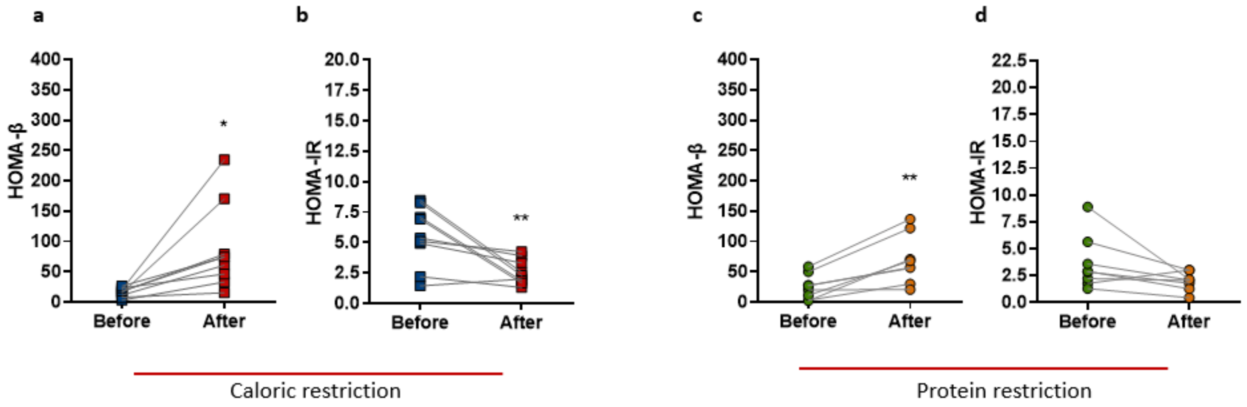

Supplementary Figure S4. Representation of HOMA-Beta and HOMA-IR after removal of 2 outlier from CR and PR protocols. A. HOMA-β comparing before and after caloric restriction after removal of 2 outliers (*P= .0132). B. HOMA-IR comparing before and after caloric restriction after removing 2 outliers (**P= .0090). C. HOMA-β comparing before and after protein restriction after removing 2 outliers (**P= .0025). HOMA-IR comparing before and after protein restriction after removing 2 outliers (P= .0835).

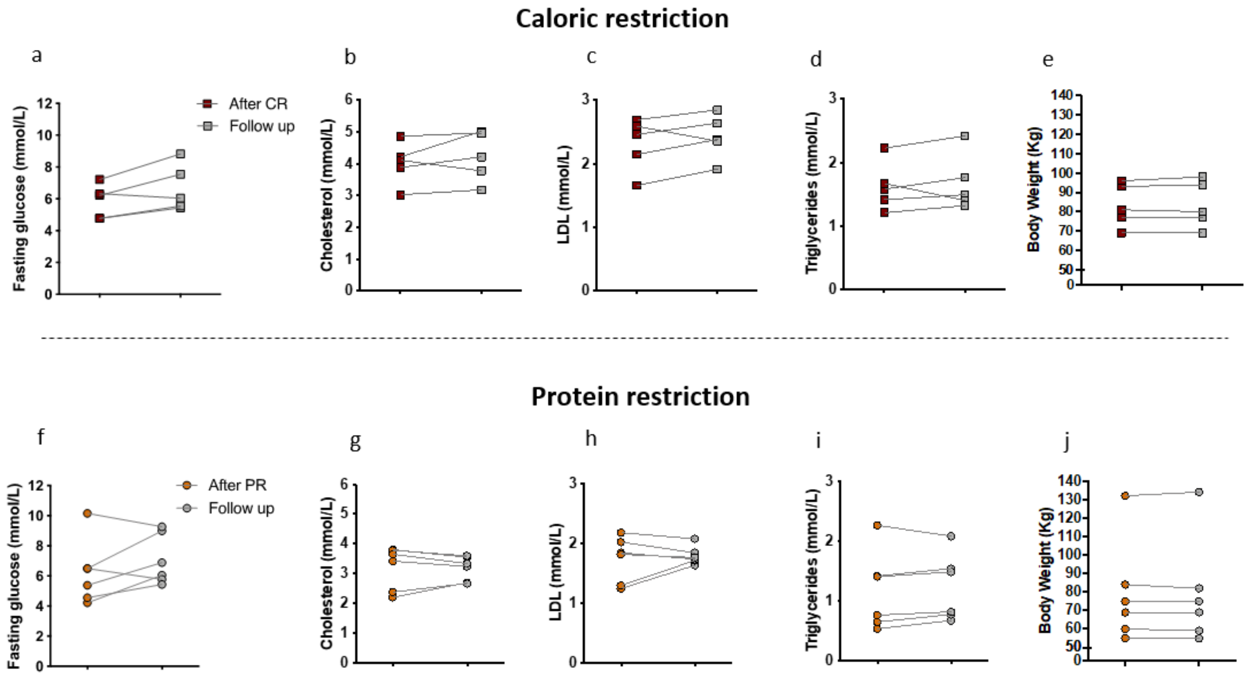

Supplementary Figure S5. Metabolic parameters after follow-up period. A-E, individuals previously assigned to the CR group. F-J, individuals previously assigned to the PR group. A, Fasting glucose levels (*P*= 0.0650). B, Total cholesterol levels (*P*= 0.3138). C, low density lipoprotein (LDL) cholesterol levels (*P*= 0.2559). D, Triglycerides levels (*P*= 0.5280). E, Body weight (*P*= 0.4766). F, Fasting glucose levels (*P*= 0.1926). G, Total cholesterol levels (*P*= 0.8557). H, low density lipoprotein (LDL) cholesterol levels (*P*= 0.6038). I, Triglycerides levels (*P*= 0.3017). J, Body weight (*P*= 0.7711). Data are presented in dot-plot format. CR (n=5). PR (n=6). Paired t test was performed.

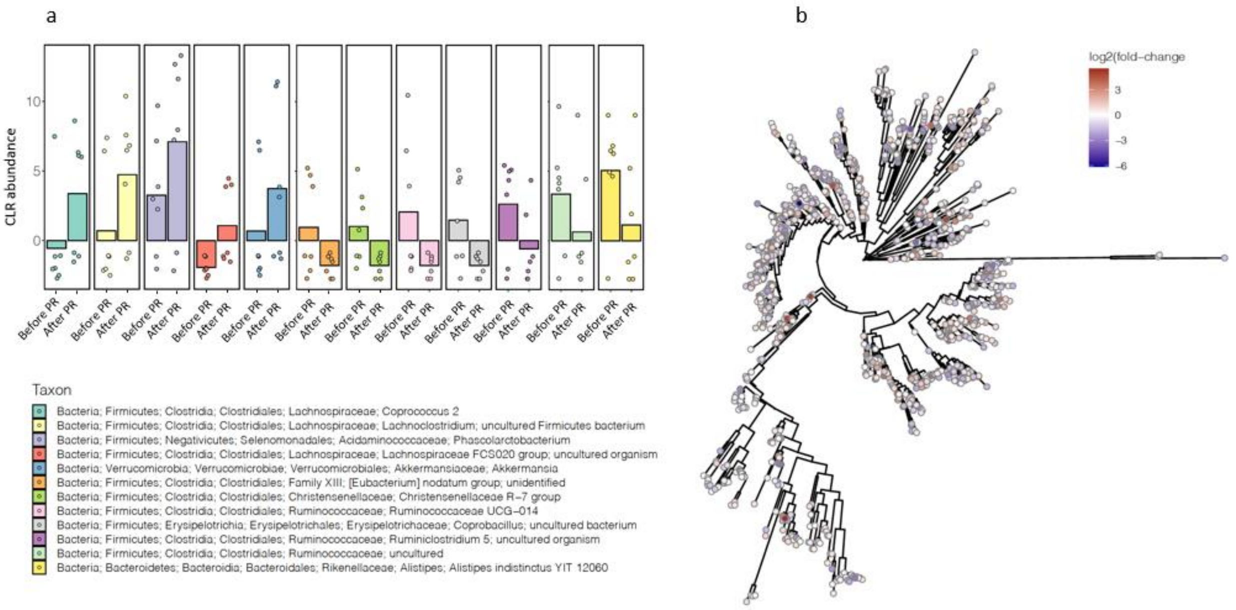

Supplementary Figure S6. PR has no short-term effects on gut microbiota composition. A. Gut microbiota composition. Relative differential abundance of bacteria (Log2). B. Bacterial phylogenetic tree construction.

A)

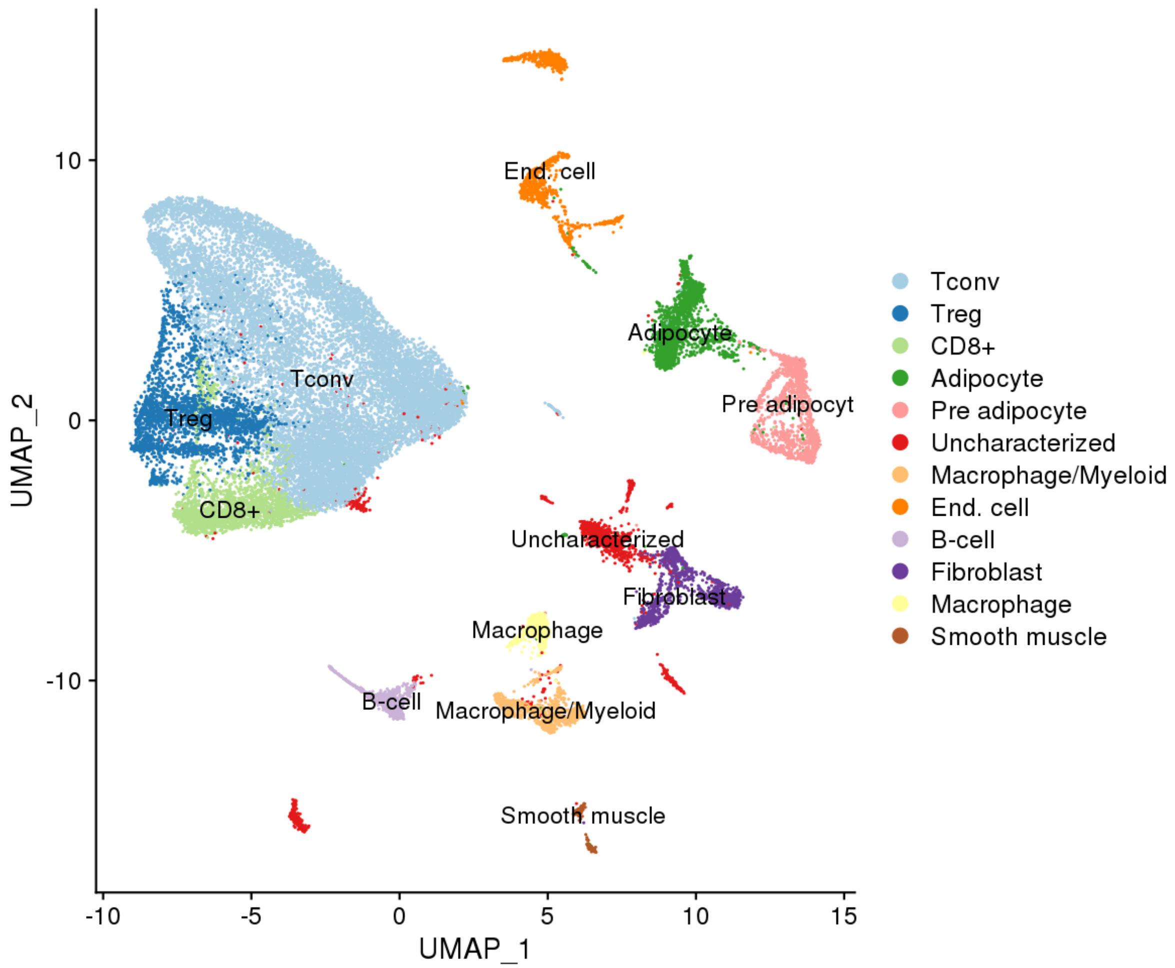
End. cell
Adipocyte
Tconv
Treg
Pre adipocyt
CD8+
Uncharacterized
Fibroblast
Macrophage
B-cell
Macrophage/Myeloid
Smooth muscle
10
0
-10
-10
-5
0
5
10
15
UMAP_2
UMAP_1
Tconv
Treg
CD8+
Adipocyte
Pre adipocyte
Uncharacterized
Macrophage/Myeloid
End. cell
B-cell
Fibroblast
Macrophage
Smooth muscle

B)

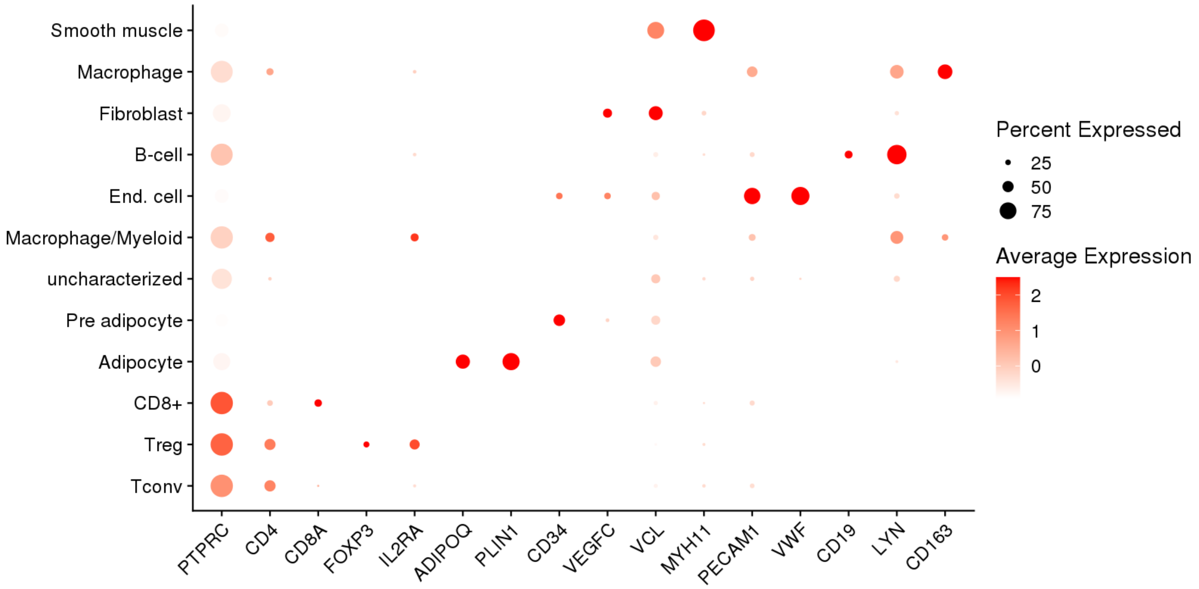

Supplementary Figure S7. Populations in the brown adipose tissue from Sun et al, 2020. **A**) Clusters were annotated according to their expression of marker genes and to expected cell types from the original paper (Sun et al., 2020). Four clusters not expressing any of the marker genes used in this work were tagged as uncharacterized. **B**) The expression of marker genes was verified in the processed snRNASeq dataset to identify and annotate populations in the adipose tissue using Seurat 4 package. The depicted markers were used to identify the populations of interest.

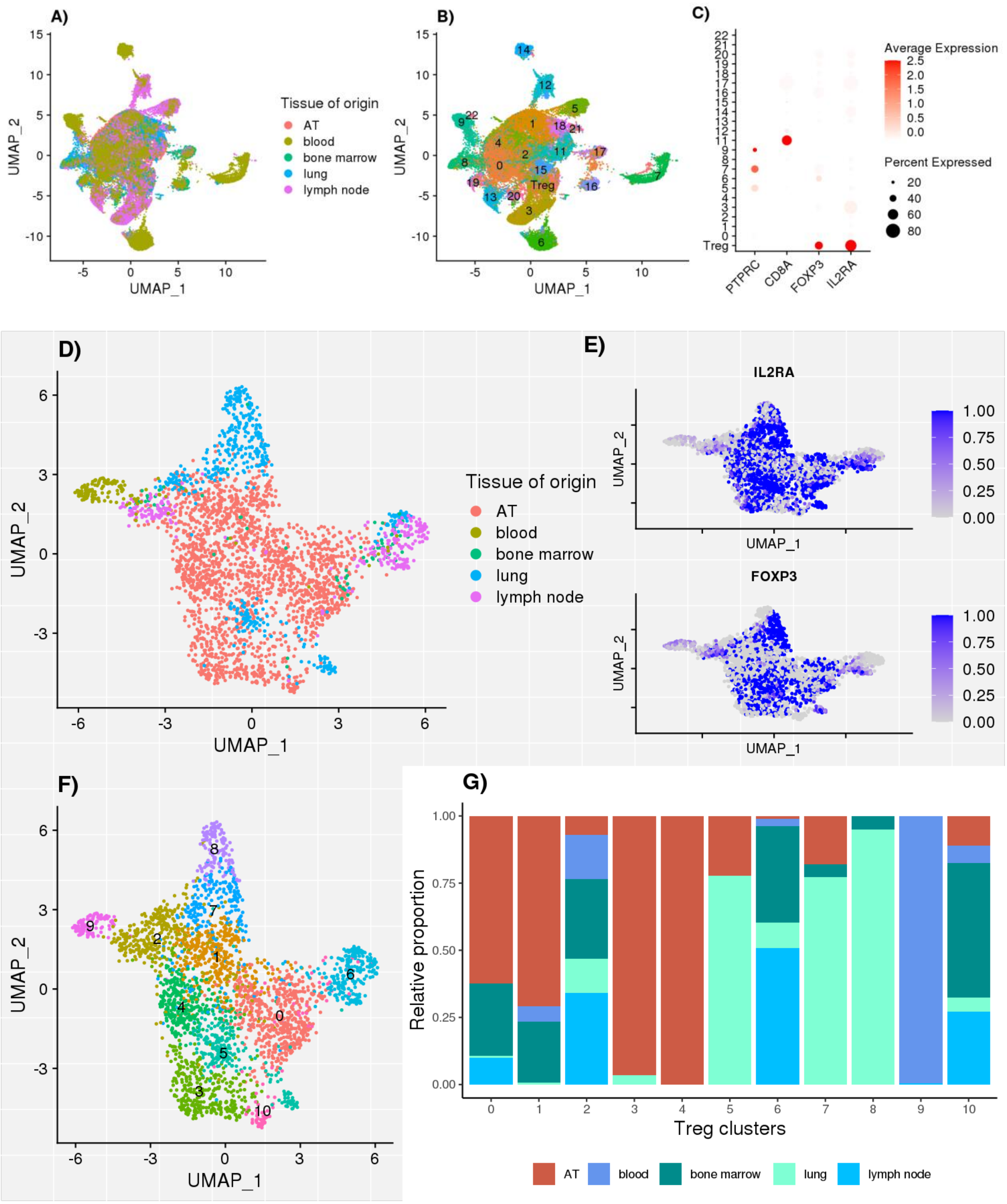

Supplementary Figure S8. Integration at the single-cell level of blood (Szabo et al., 2019) and brown adipose tissue cells (Sun et al., 2020) to isolate blood Treg cells. **A**) UMAP visualization of clusters after data integration of two datasets containing cells from five different tissues evidencing the overlap among tissues and datasets. **B**) UMAP evidencing the Treg cluster after its identification using marker genes (**C**), as evidenced in the dotplots. **D**) UMAP visualization of Treg

subclusters evidencing differences regarding the tissue of origin. **E**) Expression of marker genes within the Treg clusters. **F**) UMAP representation of 11 Treg population subclusters. **G**) Contribution of the tissue of origin to the formation of Treg subclusters. Cluster 9 was later used as signatures of Blood Tregs. Bars indicate the relative proportion of cells in each cluster.

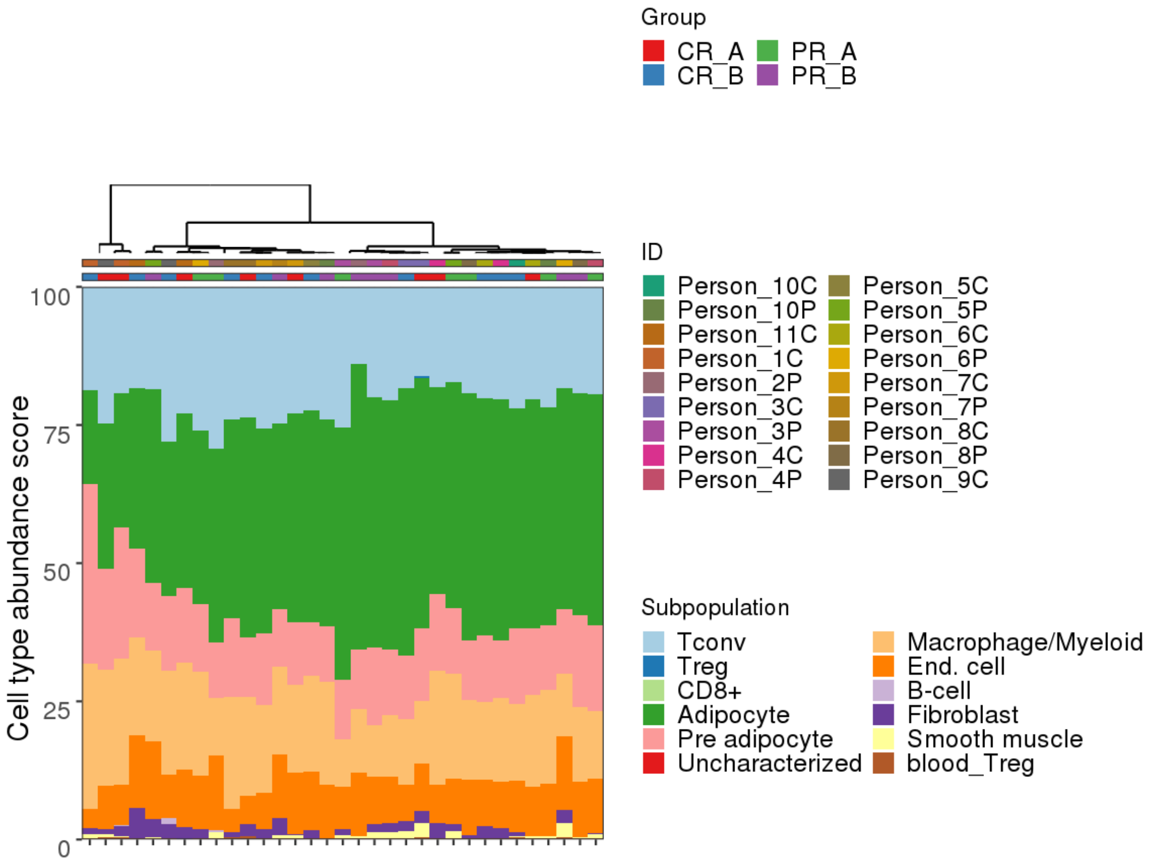

Supplementary Figure S9. Deconvolution of bulk AT RNASeq into cell types using a scRNASeq signature matrix of AT-cells and blood Treg**.** Cell types in a scRNASeq dataset of AT were clustered and annotated accordingly (Sun et al., 2019) and integrated to a blood-specific Treg cluster from a different dataset (Szabo et al., 2019) to serve as a reference signature to deconvolute bulk RNASeq samples from AT. The bars colors represent the relative quantity of each subpopulation estimated for all the different samples (ID). The top labels on the barplot indicate patient and group, respectively. Ward.D distance was used for column clustering. Deconvolution was performed using CIBERSORTx and only results with $p < 0.1$ are shown.

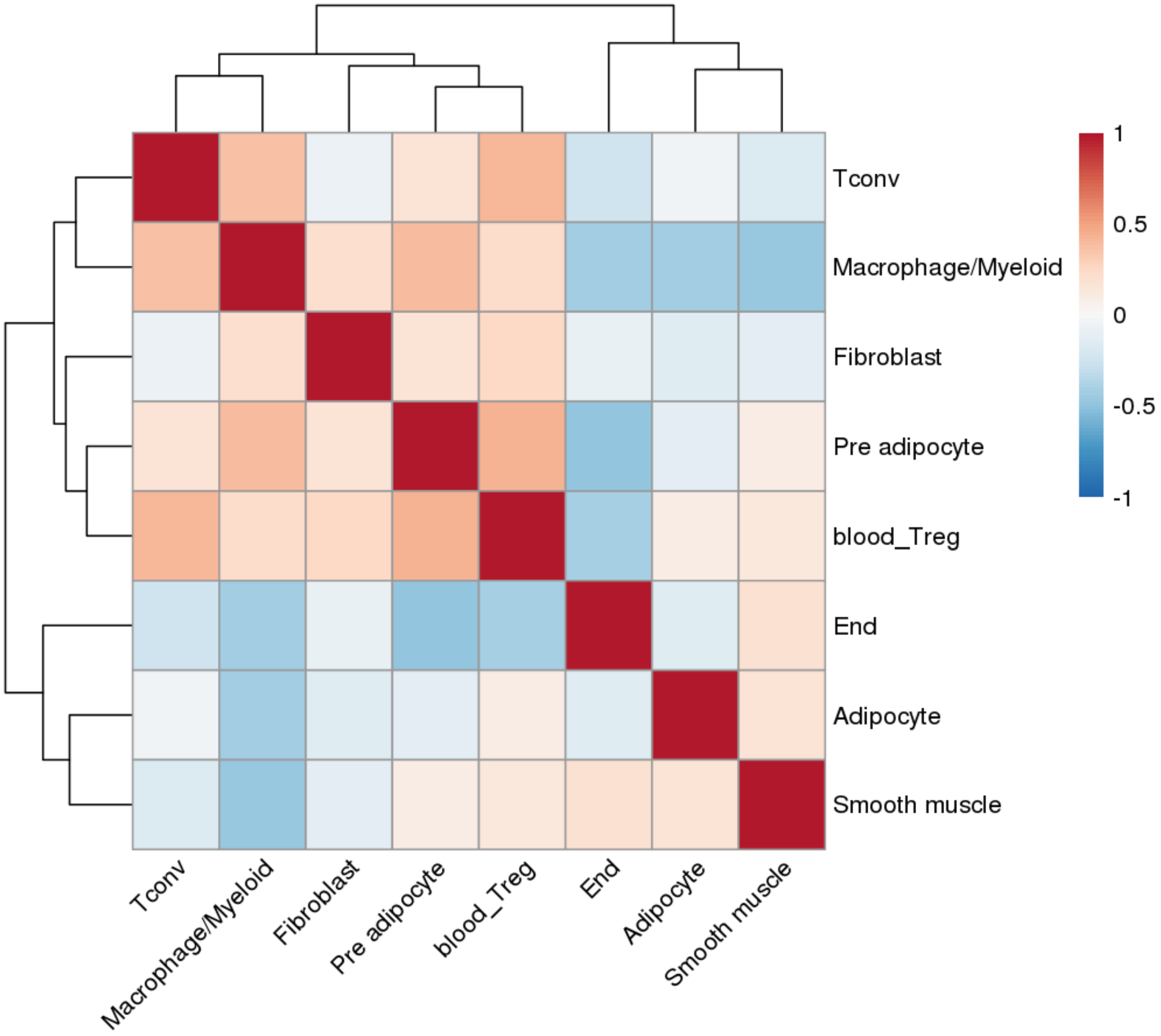

Supplementary Figure S10. Co-occurrence of cell types in the Adipose tissue. Correlation matrix of cell types proportions estimated in the AT. Results from the four groups were aggregated to investigate general tendencies in the AT. Correlations were filtered for a $p < 0.05$. Colors represent the Pearson correlation coefficient.

SUPPLEMENTAL METHODS

RNA Seq

Briefly, RNA was isolated using Trizol (Thermofisher) followed by purification with RNeasy mini kit (Qiagen). Libraries were prepared using the Illumina TruSeq Stranded total RNA Gold protocol (Illumina). Total RNA was depleted of rRNA, fragmented and converted to cDNA. cDNA was subsequently adenylated to prime for adaptor ligation and cleaned using AMPure beads. DNA fragments were amplified using PCR and subject to a final cleanup. Libraries were quality controlled using a Bioanalyzer instrument (Agilent Technologies).

Libraries were subjected to 51-bp paired-end sequencing on a NovaSeq 6000 system (Illumina). A total of 1.381 billion reads were generated. STAR v. 2.7.2b [1] was used to align reads onto the ENSEMBL 98 hg38 human genome assembly, with the GENCODE 32 gene model, both retrieved on 2019-10-07. Following mapping, reads were summarized onto genes by featureCounts v. 1.6.4 [2]. Fragments were only counted where both ends of the fragment were aligned. Six samples were excluded as they were found to cluster by themselves in an MDS plot, and had skewed density plots. Differential expression testing was performed using the R package Limma [3]. Prior to analysis, data was transformed with the voomWithQualityWeights function, followed by duplicateCorrelation and a second round of voomWithQualityWeights to reduce variance between participants [4]. All of these functions are found in the Limma package. Data were analyzed using the model ~Group. Gene set test was performed using CAMERA through Limma [5].

Single-cell processing and integration

Empty droplets were filtered by eliminating low quality data (gene count =< than 1,000, nFeature_RNA <4,000, percent.mt <25, nCount_RNA < 10,000). Data were normalized with the SCTransform workflow using a resolution of 0.4 prior to cluster annotation.

Pre-processed count matrix of the T-cell dataset from Szabo et al., 2019 [6] was retrieved at GEO database under accession number GSE126030 (https://www.ncbi.nlm.nih.gov/geo/query/acc.cgi?acc=GSE126030). Data were also pre-processed with the Seurat v4 package to remove low quality cells (nFeature_RNA <2,000, nCount_RNA < 7,000) and normalized data through the SCTransform. Integration with the processed BAT dataset was performed using 3,000 anchor genes. Besides, the tissue of origin was used to correct for the possible batches during the integration (split.by = "tissue" in the function SplitObject) since there are cells from 4 tissues (blood, bone-marrow, lung, lymph-node) in the T-cell dataset and from 1 tissue in the BAT. Expression of IL2RA and FOXP3 was used to identify the Treg cluster.

Fecal sampling and 16S rRNA Sequencing

Total gDNA was extracted from 180-200 mg sample using NucleoSpin® Soil Kit (Macherey-Nagel) following the manufacturer’s instructions. In brief, samples were lysed into 2 mL tubes containing ceramic beads, SL1 lysis buffer and SX enhancer buffer using TissueLyser II (Qiagen)

for two rounds of 3 min, 30 times/sec frequency, and 10 min incubation at room temperature. Samples were centrifuged at 13,000 *g*, the supernatant collected, and the impurities precipitated by adding SL3 buffer. After new centrifugation at 13,000 *g*, supernatants were filtered using SB buffer for DNA biding, followed by one wash in SW1 buffer, two washes in SW2 buffer and DNA elution in 30 µL of SE buffer. Samples were stored at -20°C.

Total gDNA was measured in Nanodrop2000 (Thermo Scientific), and quality was assessed through fluorimetric assay (Qubit - Invitrogen). All samples were shipped to BGI Co., Ltd (Hong-Kong, China) for Meta 16S V4 region library preparation and PE250 sequencing using the Illumina HiSeq2500 (Illumina) system with 10K reads per sample.

Metagenomic Analysis

A feature table was created for each cohort, and analyses for alpha- and beta-diversity metrics were performed. Since some diversity metrics require phylogenetic relationships between the features, a rooted phylogenetic tree using MAFFT alignment [7] and fasttree2 pipeline [8] was constructed. α-diversity was calculated using Shannon diversity index [9], Faith Phylogenetic Diversity [10], Observed OTUs, and Pielou's evenness metrics [11]. ß-diversity was compared using Bray-Curtis, Jaccard, weighted, and unweighted UniFrac distance matrices [12, 13]. Data normalization was done through rarefaction for keeping the same sequencing depth of all samples. ß-diversity results were visualized on PCoA plots for each distance matrix, and data clustering patterns were investigated and tested using PERMANOVA and PERMDISP [14].

Differentially abundant features between two components (before and after protein restriction cohorts) were identified using ANCOM and ALDEX in QIIME [15, 16].

References

[1] Dobin A, Davis CA, Schlesinger F, et al. (2013) STAR: ultrafast universal RNA-seq aligner. Bioinformatics 29(1): 15-21. 10.1093/bioinformatics/bts635

[2] Liao Y, Smyth GK, Shi W (2014) featureCounts: an efficient general purpose program for assigning sequence reads to genomic features. Bioinformatics 30(7): 923-930. 10.1093/bioinformatics/btt656

[3] Ritchie ME, Phipson B, Wu D, et al. (2015) limma powers differential expression analyses for RNA-sequencing and microarray studies. Nucleic acids research 43(7): e47. 10.1093/nar/gkv007

[4] Liu R, Holik AZ, Su S, et al. (2015) Why weight? Modelling sample and observational level variability improves power in RNA-seq analyses. Nucleic acids research 43(15): e97. 10.1093/nar/gkv412

[5] Wu D, Smyth GK (2012) Camera: a competitive gene set test accounting for inter-gene correlation. Nucleic acids research 40(17): e133. 10.1093/nar/gks461

[6] Szabo PA, Levitin HM, Miron M, et al. (2019) Single-cell transcriptomics of human T cells reveals tissue and activation signatures in health and disease. Nature communications 10(1): 4706. 10.1038/s41467-019-12464-3

[7] Katoh K, Misawa K, Kuma K, Miyata T (2002) MAFFT: a novel method for rapid multiple sequence alignment based on fast Fourier transform. Nucleic Acids Res 30(14): 3059-3066. 10.1093/nar/gkf436

[8] Price MN, Dehal PS, Arkin AP (2010) FastTree 2--approximately maximum-likelihood trees for large alignments. PLoS One 5(3): e9490. 10.1371/journal.pone.0009490

[9] Spellerberg IF, Fedor PJ (2003) A tribute to Claude Shannon (1916–2001) and a plea for more rigorous use of species richness, species diversity and the 'Shannon–Wiener' Index. Global Ecology and Biogeography 12: 177-179. https://doi.org/10.1046/j.1466-822X.2003.00015.x

[10] Faith DP (1992) Conservation evaluation and phylogenetic diversity. Biological Conservation 61(1): 1-10. https://doi.org/10.1016/0006-3207(92)91201-3

[11] Pielou EC (1966) The measurement of diversity in different types of biological collections. Biological Conservation 31: 131-144. https://doi.org/10.1016/0022-5193(66)90013-0

[12] Lozupone C, Knight R (2005) UniFrac: a new phylogenetic method for comparing microbial communities. Appl Environ Microbiol 71(12): 8228-8235. 10.1128/AEM.71.12.8228-8235.2005

[13] Lozupone CA, Hamady M, Kelley ST, Knight R (2007) Quantitative and qualitative beta diversity measures lead to different insights into factors that structure microbial communities. Appl Environ Microbiol 73(5): 1576-1585. 10.1128/AEM.01996-06

[14] Anderson MJ (2017) Permutational Multivariate Analysis of Variance (PERMANOVA). Willey Stats Ref: Statistics Reference Online. https://doi.org/10.1002/9781118445112.stat07841
[15] Mandal S, Van Treuren W, White RA, Eggesbø M, Knight R, Peddada SD (2015) Analysis of composition of microbiomes: a novel method for studying microbial composition. Microb Ecol Health Dis 26: 27663. 10.3402/mehd.v26.27663
[16] Fernandes AD, Macklaim JM, Linn TG, Reid G, Gloor GB (2013) ANOVA-like differential expression (ALDEx) analysis for mixed population RNA-Seq. PLoS One 8(7): e67019. 10.1371/journal.pone.0067019
